# 2-Hydr­oxy-16-[(*E*)-4-hydr­oxy-3-methoxy­benzyl­idene]-13-(4-hydr­oxy-3-methoxy­phen­yl)-11-methyl-1,11-diaza­penta­cyclo­[12.3.1.0^2,10^.0^3,8^.0^10,14^]octa­deca-3(8),4,6-triene-9,15-dione

**DOI:** 10.1107/S1600536810017216

**Published:** 2010-05-15

**Authors:** Raju Suresh Kumar, Hasnah Osman, Mohamed Ashraf Ali, Madhukar Hemamalini, Hoong-Kun Fun

**Affiliations:** a School of Chemical Sciences, Universiti Sains Malaysia, 11800 USM, Penang, Malaysia; b Institute for Research in Molecular Medicine, Universiti Sains Malaysia, 11800 USM, Penang, Malaysia; c X-ray Crystallography Unit, School of Physics, Universiti Sains Malaysia, 11800 USM, Penang, Malaysia

## Abstract

In the title compound, C_32_H_30_N_2_O_7_, the piperidone ring adopts a chair conformation and the five-membered ring of the dihydro­indenone ring system adopts an envelope conformation. Intra­molecular O—H⋯N and C—H⋯O hydrogen bonds occur. The dihedral angle between the two hydr­oxy-subsituted methoxy­phenyl rings is 71.12 (5)°. In the crystal structure, mol­ecules are connected by inter­molecular O—H⋯O hydrogen bonds, forming layers parallel to (001). These layers are further connected by C—H⋯O hydrogen bonds, forming a three-dimensional network.

## Related literature

For details of 1,3-dipolar cyclo­addition, see: Padwa (1984 ▶). For applications of pyrrolidines, see: Dalko & Moisan (2004 ▶); Seayad & List (2005 ▶); Natarajan *et al.* (2006 ▶); O’Hagan (2000 ▶). For puckering parameters, see: Cremer & Pople (1975 ▶). For the stability of the temperature controller used in the data collection, see: Cosier & Glazer (1986 ▶).
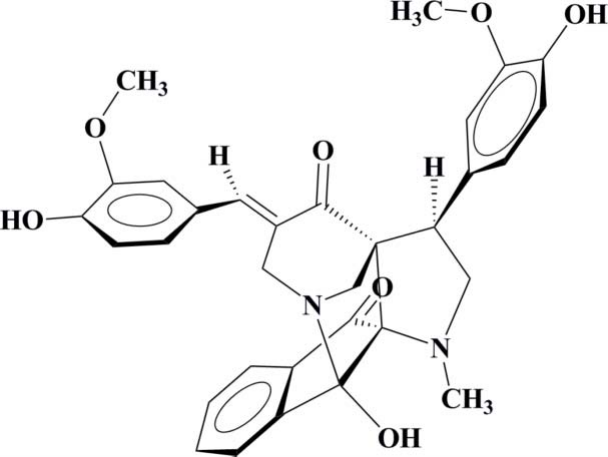


## Experimental

### 

#### Crystal data

C_32_H_30_N_2_O_7_
*M* *_r_* = 554.58
Orthorhombic, 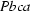

*a* = 14.7989 (11) Å
*b* = 15.4918 (11) Å
*c* = 22.9079 (17) Å
*V* = 5251.9 (7) Å^3^
*Z* = 8
Mo *K*α radiation
μ = 0.10 mm^−1^
*T* = 100 K
0.39 × 0.39 × 0.31 mm

#### Data collection

Bruker APEXII DUO CCD area-detector diffractometer
Absorption correction: multi-scan (*SADABS*; Bruker, 2009 ▶) *T*_min_ = 0.962, *T*_max_ = 0.969
130228 measured reflections
9672 independent reflections
7897 reflections with *I* > 2σ(*I*)
*R*_int_ = 0.048

#### Refinement

*R*[*F* ^2^ > 2σ(*F* ^2^)] = 0.051
*wR*(*F* ^2^) = 0.145
*S* = 1.09
9672 reflections
385 parameters
H atoms treated by a mixture of independent and constrained refinement
Δρ_max_ = 0.59 e Å^−3^
Δρ_min_ = −0.25 e Å^−3^

### 

Data collection: *APEX2* (Bruker, 2009 ▶); cell refinement: *SAINT* (Bruker, 2009 ▶); data reduction: *SAINT*; program(s) used to solve structure: *SHELXTL* (Sheldrick, 2008 ▶); program(s) used to refine structure: *SHELXTL*; molecular graphics: *SHELXTL*; software used to prepare material for publication: *SHELXTL* and *PLATON* (Spek, 2009 ▶).

## Supplementary Material

Crystal structure: contains datablocks global, I. DOI: 10.1107/S1600536810017216/is2545sup1.cif

Structure factors: contains datablocks I. DOI: 10.1107/S1600536810017216/is2545Isup2.hkl

Additional supplementary materials:  crystallographic information; 3D view; checkCIF report

## Figures and Tables

**Table 1:** Hydrogen-bond geometry (Å, °)

*D*—H⋯*A*	*D*—H	H⋯*A*	*D*⋯*A*	*D*—H⋯*A*
O5—H1*O*5⋯O2^i^	0.89 (2)	1.77 (2)	2.6554 (14)	173 (2)
O7—H1*O*7⋯O4^ii^	0.88 (3)	2.13 (3)	2.8720 (13)	143 (2)
O2—H1*O*2⋯N2	0.92 (3)	1.91 (2)	2.5987 (13)	131 (2)
C1—H1*A*⋯O7^iii^	0.97	2.49	3.3771 (16)	151
C13—H13*A*⋯O7^iv^	0.93	2.57	3.3237 (17)	138
C27—H27*A*⋯O1	0.93	2.52	3.0998 (15)	121
C32—H32*A*⋯O3^v^	0.96	2.40	3.1819 (17)	139

Symmetry codes: (i) 
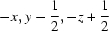
; (ii) 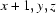; (iii) 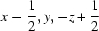; (iv) 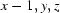; (v) 
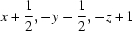
.

**Fig. 1.**
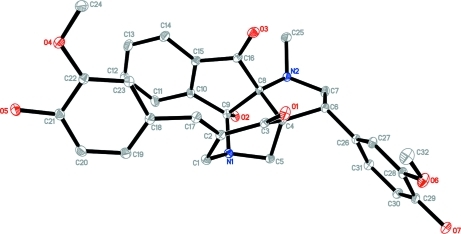
The asymmetric unit of the title compound, showing 30% probability displacement ellipsoids and the atom-numbering scheme (H atoms are omitted for clarity).

**Fig. 2.**
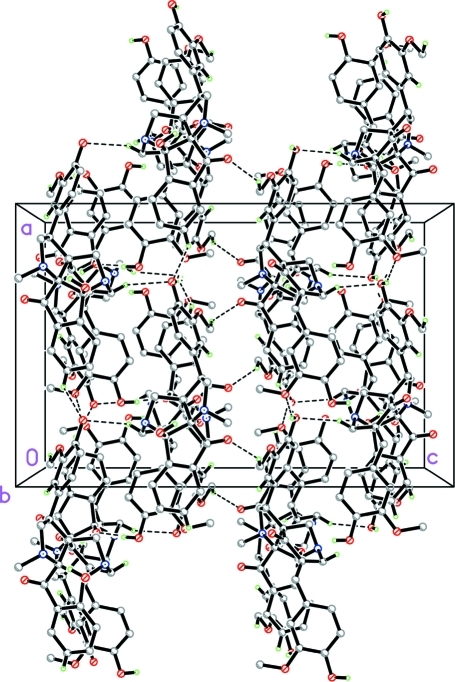
The crystal packing of the title compound, showing hydrogen-bonded (dashed lines) network. H atoms are not involving the hydrogen bond interactions are omitted for clarity.

## supplementary crystallographic information

### Comment

1,3-Dipolar cycloadditions represent one of the most versatile tools for the construction of five-membered heterocycles (Padwa, 1984). A diverse array of biologically active molecules contain substituted pyrrolidine cores (O'Hagan, 2000). Pyrrolidines are important building blocks in organic synthesis, and in the past years have emerged as privileged organocatalysts (Dalko & Moisan, 2004; Seayad & List, 2005). Compounds containing the piperidine subunit act as excellent inhibitors of p38 activity and TNF–α release (Natarajan *et al.*, 2006) and consequently, the efficient preparation of these heterocycles has received significant attention.

The molecular structure of the title compound is shown in Fig. 1. The piperidone (N1/C1–C5) ring adopts a chair conformation [Q = 0.6146 (12) Å, Θ = 145.39 (11)°, φ = 120.0 (2)° ; Cremer & Pople, 1975]. The pyrrolidine ring (N2/C4/C6–C8) adopts an envelope conformation [puckering parameters Q = 0.3365 (12) Å, φ = 84.3 (2)°]. The five membered ring of the ninhydrin system adopts an envelope conformation and its flap atom C8 deviates from the mean plane formed by other atoms, C9–C16, by 0.0702 (12) Å. The dihedral angle between the two hydroxy subsituted methoxyphenyl rings (C18–C23 and C26–C31) is 71.12 (5)°.

In the crystal packing (Fig. 2), molecules are connected by intermolecular O2—H1O5···O2, O7—H1O7···O4, C1—H1A···O7, C13—H13A···O7 and C32—H32A···O3 hydrogen bonds to form a three–dimensional network.

### Experimental

A mixture of 3,5-bis[(*E*)–(4-hydroxy-3-methoxyphenyl)methylidene] tetrahydro–4(1*H*)–pyridinone (0.100 g, 0.272 mmol), ninhydrin (0.049 g, 0.272 mmol), and sarcosine (0.024 g, 0.272 mmol) were dissolved in methanol (10 mL) and refluxed for 1 hour. After completion of the reaction as evident from TLC, the mixture was poured into water (50 mL). The precipitated solid was filtered and washed with water to afford the product which was recrystallized from petrolium ether-ethyl acetate mixture (1:1) to reveal the title compound as yellow crystals.

### Refinement

Atoms H1O2, H1O5 and H1O7 were located in a difference Fourier map and refined freely. The remaining H atoms were positioned geometrically (C–H = 0.93–0.98 Å) and were refined using a riding model, with *U*_iso_(H) = 1.2 or 1.5*U*_eq_(C). A rotating group model was applied to the methyl groups.

### Crystal data

**Table:**

C_32_H_30_N_2_O_7_	*F*(000) = 2336
*M**_r_* = 554.58	*D*_x_ = 1.403 Mg m^−^^3^
Orthorhombic, *P**b**c**a*	Mo *K*α radiation, λ = 0.71073 Å
Hall symbol: -P 2ac 2ab	Cell parameters from 9860 reflections
*a* = 14.7989 (11) Å	θ = 2.3–32.6°
*b* = 15.4918 (11) Å	µ = 0.10 mm^−^^1^
*c* = 22.9079 (17) Å	*T* = 100 K
*V* = 5251.9 (7) Å^3^	Block, yellow
*Z* = 8	0.39 × 0.39 × 0.31 mm

### Data collection

**Table:**

Bruker APEXII DUO CCD area-detector diffractometer	9672 independent reflections
Radiation source: fine-focus sealed tube	7897 reflections with *I* > 2σ(*I*)
graphite	*R*_int_ = 0.048
φ and ω scans	θ_max_ = 32.7°, θ_min_ = 1.8°
Absorption correction: multi-scan (*SADABS*; Bruker, 2009)	*h* = −22→19
*T*_min_ = 0.962, *T*_max_ = 0.969	*k* = −23→23
130228 measured reflections	*l* = −33→34

### Refinement

**Table:**

Refinement on *F*^2^	Primary atom site location: structure-invariant direct methods
Least-squares matrix: full	Secondary atom site location: difference Fourier map
*R*[*F*^2^ > 2σ(*F*^2^)] = 0.051	Hydrogen site location: inferred from neighbouring sites
*wR*(*F*^2^) = 0.145	H atoms treated by a mixture of independent and constrained refinement
*S* = 1.09	*w* = 1/[σ^2^(*F*_o_^2^) + (0.0657*P*)^2^ + 3.1726*P*] where *P* = (*F*_o_^2^ + 2*F*_c_^2^)/3
9672 reflections	(Δ/σ)_max_ = 0.001
385 parameters	Δρ_max_ = 0.59 e Å^−^^3^
0 restraints	Δρ_min_ = −0.25 e Å^−^^3^

### Special details

**Table:**

Experimental. The crystal was placed in the cold stream of an Oxford Cryosystems Cobra open-flow nitrogen cryostat (Cosier & Glazer, 1986) operating at 100.0 (1) K.
Geometry. All s.u.'s (except the s.u. in the dihedral angle between two l.s. planes) are estimated using the full covariance matrix. The cell s.u.'s are taken into account individually in the estimation of s.u.'s in distances, angles and torsion angles; correlations between s.u.'s in cell parameters are only used when they are defined by crystal symmetry. An approximate (isotropic) treatment of cell s.u.'s is used for estimating s.u.'s involving l.s. planes.
Refinement. Refinement of F^2^ against ALL reflections. The weighted R-factor wR and goodness of fit S are based on F^2^, conventional R-factors R are based on F, with F set to zero for negative F^2^. The threshold expression of F^2^ > 2σ(F^2^) is used only for calculating R-factors(gt) etc. and is not relevant to the choice of reflections for refinement. R-factors based on F^2^ are statistically about twice as large as those based on F, and R- factors based on ALL data will be even larger.

### Fractional atomic coordinates and isotropic or equivalent isotropic displacement parameters (Å^2^)

**Table:**

	*x*	*y*	*z*	*U*_iso_*/*U*_eq_	
O1	0.30327 (6)	−0.18897 (6)	0.41100 (4)	0.02184 (18)	
O2	0.19750 (6)	0.13975 (5)	0.33372 (4)	0.01979 (17)	
O3	0.16366 (7)	−0.05592 (6)	0.48344 (4)	0.02408 (19)	
O4	−0.17817 (6)	−0.29718 (6)	0.38154 (4)	0.02209 (18)	
O5	−0.21109 (7)	−0.28735 (7)	0.27082 (5)	0.0265 (2)	
O6	0.62083 (6)	−0.24385 (6)	0.41434 (5)	0.02473 (19)	
O7	0.72935 (6)	−0.13939 (6)	0.35393 (4)	0.02204 (18)	
N1	0.22581 (7)	0.00074 (6)	0.29585 (4)	0.01670 (18)	
N2	0.27389 (7)	0.09389 (6)	0.43104 (4)	0.01600 (17)	
C1	0.18714 (8)	−0.08658 (7)	0.28648 (5)	0.0184 (2)	
H1A	0.2167	−0.1127	0.2531	0.022*	
H1B	0.1235	−0.0807	0.2771	0.022*	
C2	0.19647 (8)	−0.14690 (7)	0.33836 (5)	0.0168 (2)	
C3	0.27292 (8)	−0.13242 (7)	0.37943 (5)	0.01594 (19)	
C4	0.30624 (7)	−0.03950 (7)	0.38134 (5)	0.01382 (18)	
C5	0.31885 (8)	−0.00938 (8)	0.31723 (5)	0.0174 (2)	
H5A	0.3514	0.0449	0.3155	0.021*	
H5B	0.3513	−0.0523	0.2946	0.021*	
C6	0.38290 (8)	−0.02131 (7)	0.42424 (5)	0.01551 (19)	
H6A	0.3693	−0.0526	0.4604	0.019*	
C7	0.37136 (8)	0.07523 (8)	0.43694 (6)	0.0204 (2)	
H7A	0.3919	0.0886	0.4761	0.025*	
H7B	0.4060	0.1094	0.4094	0.025*	
C8	0.22873 (7)	0.02205 (7)	0.40137 (5)	0.01406 (18)	
C9	0.18191 (8)	0.05074 (7)	0.34284 (5)	0.01539 (19)	
C10	0.08268 (8)	0.03156 (7)	0.35136 (5)	0.0175 (2)	
C11	0.01111 (9)	0.04857 (9)	0.31373 (6)	0.0237 (2)	
H11A	0.0207	0.0761	0.2782	0.028*	
C12	−0.07522 (9)	0.02326 (10)	0.33061 (7)	0.0294 (3)	
H12A	−0.1239	0.0349	0.3062	0.035*	
C13	−0.09026 (9)	−0.01932 (10)	0.38355 (7)	0.0281 (3)	
H13A	−0.1484	−0.0366	0.3935	0.034*	
C14	−0.01931 (9)	−0.03585 (8)	0.42110 (6)	0.0223 (2)	
H14A	−0.0289	−0.0638	0.4565	0.027*	
C15	0.06727 (8)	−0.00943 (7)	0.40453 (5)	0.0174 (2)	
C16	0.15262 (8)	−0.02051 (7)	0.43663 (5)	0.0167 (2)	
C17	0.13529 (8)	−0.20654 (7)	0.35528 (5)	0.0180 (2)	
H17A	0.1508	−0.2383	0.3882	0.022*	
C18	0.04812 (8)	−0.22830 (7)	0.32917 (5)	0.0180 (2)	
C19	0.02938 (9)	−0.22466 (8)	0.26938 (6)	0.0216 (2)	
H19A	0.0746	−0.2094	0.2432	0.026*	
C20	−0.05737 (9)	−0.24393 (8)	0.24901 (6)	0.0228 (2)	
H20A	−0.0695	−0.2408	0.2092	0.027*	
C21	−0.12563 (8)	−0.26768 (8)	0.28732 (5)	0.0197 (2)	
C22	−0.10679 (8)	−0.27274 (7)	0.34744 (5)	0.0173 (2)	
C23	−0.02099 (8)	−0.25368 (7)	0.36762 (5)	0.0173 (2)	
H23A	−0.0088	−0.2577	0.4073	0.021*	
C24	−0.15933 (9)	−0.31881 (9)	0.44084 (6)	0.0241 (2)	
H24A	−0.2137	−0.3384	0.4594	0.036*	
H24B	−0.1369	−0.2688	0.4609	0.036*	
H24C	−0.1147	−0.3638	0.4421	0.036*	
C25	0.23206 (9)	0.12884 (9)	0.48352 (6)	0.0251 (3)	
H25A	0.1692	0.1397	0.4762	0.038*	
H25B	0.2615	0.1818	0.4941	0.038*	
H25C	0.2379	0.0880	0.5148	0.038*	
C26	0.47590 (7)	−0.04947 (7)	0.40435 (5)	0.01503 (19)	
C27	0.50398 (8)	−0.13305 (7)	0.41924 (5)	0.0173 (2)	
H27A	0.4659	−0.1684	0.4409	0.021*	
C28	0.58767 (8)	−0.16339 (7)	0.40201 (5)	0.0175 (2)	
C29	0.64600 (8)	−0.11047 (8)	0.36990 (5)	0.0172 (2)	
C30	0.61868 (8)	−0.02804 (8)	0.35443 (5)	0.0192 (2)	
H30A	0.6569	0.0072	0.3328	0.023*	
C31	0.53351 (8)	0.00208 (7)	0.37139 (5)	0.0182 (2)	
H31A	0.5152	0.0572	0.3605	0.022*	
C32	0.55997 (10)	−0.30334 (9)	0.44075 (7)	0.0302 (3)	
H32A	0.5886	−0.3587	0.4441	0.045*	
H32B	0.5067	−0.3085	0.4171	0.045*	
H32C	0.5436	−0.2829	0.4789	0.045*	
H1O5	−0.2099 (15)	−0.3091 (16)	0.2347 (10)	0.044 (6)*	
H1O7	0.7311 (17)	−0.1945 (17)	0.3626 (11)	0.052 (7)*	
H1O2	0.2281 (16)	0.1577 (16)	0.3664 (11)	0.049 (6)*	

### Atomic displacement parameters (Å^2^)

**Table:**

	*U*^11^	*U*^22^	*U*^33^	*U*^12^	*U*^13^	*U*^23^
O1	0.0183 (4)	0.0159 (4)	0.0313 (5)	0.0002 (3)	−0.0017 (3)	0.0062 (3)
O2	0.0250 (4)	0.0128 (3)	0.0216 (4)	−0.0027 (3)	−0.0060 (3)	0.0032 (3)
O3	0.0225 (4)	0.0266 (4)	0.0231 (4)	0.0019 (3)	0.0039 (3)	0.0080 (3)
O4	0.0167 (4)	0.0276 (4)	0.0219 (4)	0.0001 (3)	0.0013 (3)	0.0012 (3)
O5	0.0192 (4)	0.0359 (5)	0.0242 (4)	−0.0008 (4)	−0.0032 (3)	−0.0094 (4)
O6	0.0202 (4)	0.0180 (4)	0.0360 (5)	0.0060 (3)	0.0009 (4)	0.0064 (4)
O7	0.0148 (4)	0.0233 (4)	0.0280 (4)	0.0034 (3)	0.0026 (3)	0.0001 (3)
N1	0.0174 (4)	0.0166 (4)	0.0160 (4)	−0.0023 (3)	−0.0005 (3)	0.0009 (3)
N2	0.0147 (4)	0.0148 (4)	0.0185 (4)	0.0008 (3)	−0.0015 (3)	−0.0027 (3)
C1	0.0207 (5)	0.0166 (5)	0.0178 (5)	−0.0025 (4)	−0.0009 (4)	−0.0010 (4)
C2	0.0172 (5)	0.0135 (4)	0.0198 (5)	0.0001 (4)	0.0010 (4)	−0.0012 (4)
C3	0.0148 (5)	0.0136 (4)	0.0194 (5)	−0.0003 (3)	0.0020 (4)	0.0006 (4)
C4	0.0131 (4)	0.0125 (4)	0.0159 (4)	0.0000 (3)	0.0006 (3)	0.0009 (3)
C5	0.0164 (5)	0.0189 (5)	0.0170 (5)	−0.0018 (4)	0.0014 (4)	0.0023 (4)
C6	0.0137 (4)	0.0149 (4)	0.0179 (4)	0.0011 (3)	−0.0007 (4)	0.0003 (4)
C7	0.0152 (5)	0.0171 (5)	0.0290 (6)	0.0006 (4)	−0.0022 (4)	−0.0055 (4)
C8	0.0132 (4)	0.0128 (4)	0.0161 (4)	0.0003 (3)	−0.0005 (3)	0.0009 (3)
C9	0.0162 (5)	0.0123 (4)	0.0177 (4)	−0.0015 (3)	−0.0017 (4)	0.0021 (3)
C10	0.0158 (5)	0.0159 (5)	0.0208 (5)	0.0000 (4)	−0.0023 (4)	−0.0021 (4)
C11	0.0196 (5)	0.0277 (6)	0.0238 (5)	0.0008 (4)	−0.0056 (4)	−0.0026 (5)
C12	0.0182 (6)	0.0388 (7)	0.0310 (7)	−0.0002 (5)	−0.0062 (5)	−0.0086 (6)
C13	0.0165 (5)	0.0340 (7)	0.0337 (7)	−0.0048 (5)	0.0012 (5)	−0.0092 (5)
C14	0.0178 (5)	0.0221 (5)	0.0271 (6)	−0.0030 (4)	0.0045 (4)	−0.0041 (4)
C15	0.0150 (5)	0.0153 (4)	0.0221 (5)	0.0003 (4)	0.0015 (4)	−0.0018 (4)
C16	0.0157 (5)	0.0147 (4)	0.0197 (5)	0.0008 (4)	0.0029 (4)	0.0005 (4)
C17	0.0177 (5)	0.0129 (4)	0.0235 (5)	−0.0007 (4)	0.0000 (4)	−0.0009 (4)
C18	0.0181 (5)	0.0135 (4)	0.0224 (5)	−0.0010 (4)	−0.0006 (4)	−0.0011 (4)
C19	0.0229 (6)	0.0205 (5)	0.0212 (5)	−0.0046 (4)	0.0016 (4)	−0.0039 (4)
C20	0.0263 (6)	0.0234 (5)	0.0186 (5)	−0.0029 (5)	−0.0009 (4)	−0.0037 (4)
C21	0.0187 (5)	0.0190 (5)	0.0215 (5)	0.0009 (4)	−0.0025 (4)	−0.0052 (4)
C22	0.0160 (5)	0.0148 (4)	0.0211 (5)	0.0016 (4)	0.0013 (4)	−0.0010 (4)
C23	0.0174 (5)	0.0144 (4)	0.0201 (5)	0.0009 (4)	−0.0011 (4)	−0.0001 (4)
C24	0.0236 (6)	0.0276 (6)	0.0209 (5)	0.0023 (5)	0.0024 (4)	0.0006 (5)
C25	0.0214 (6)	0.0282 (6)	0.0258 (6)	0.0013 (5)	0.0021 (5)	−0.0103 (5)
C26	0.0130 (4)	0.0142 (4)	0.0178 (4)	0.0004 (3)	−0.0005 (4)	0.0003 (3)
C27	0.0144 (5)	0.0158 (4)	0.0217 (5)	0.0007 (4)	−0.0006 (4)	0.0034 (4)
C28	0.0160 (5)	0.0152 (4)	0.0212 (5)	0.0024 (4)	−0.0024 (4)	0.0013 (4)
C29	0.0127 (4)	0.0189 (5)	0.0201 (5)	0.0006 (4)	−0.0007 (4)	−0.0018 (4)
C30	0.0170 (5)	0.0170 (5)	0.0235 (5)	−0.0020 (4)	0.0028 (4)	−0.0002 (4)
C31	0.0172 (5)	0.0136 (4)	0.0237 (5)	−0.0002 (4)	0.0012 (4)	0.0007 (4)
C32	0.0312 (7)	0.0188 (5)	0.0406 (7)	0.0033 (5)	0.0011 (6)	0.0099 (5)

### Geometric parameters (Å, °)

**Table:**

O1—C3	1.2216 (14)	C11—C12	1.391 (2)
O2—C9	1.4137 (13)	C11—H11A	0.9300
O2—H1O2	0.92 (2)	C12—C13	1.398 (2)
O3—C16	1.2154 (15)	C12—H12A	0.9300
O4—C22	1.3674 (15)	C13—C14	1.381 (2)
O4—C24	1.4266 (16)	C13—H13A	0.9300
O5—C21	1.3547 (15)	C14—C15	1.3975 (17)
O5—H1O5	0.89 (2)	C14—H14A	0.9300
O6—C28	1.3690 (14)	C15—C16	1.4717 (17)
O6—C32	1.4235 (17)	C17—C18	1.4613 (16)
O7—C29	1.3624 (14)	C17—H17A	0.9300
O7—H1O7	0.88 (3)	C18—C19	1.3987 (17)
N1—C5	1.4699 (15)	C18—C23	1.4059 (17)
N1—C9	1.4768 (15)	C19—C20	1.3982 (18)
N1—C1	1.4844 (15)	C19—H19A	0.9300
N2—C25	1.4567 (16)	C20—C21	1.3879 (18)
N2—C8	1.4653 (14)	C20—H20A	0.9300
N2—C7	1.4773 (15)	C21—C22	1.4072 (17)
C1—C2	1.5181 (17)	C22—C23	1.3831 (16)
C1—H1A	0.9700	C23—H23A	0.9300
C1—H1B	0.9700	C24—H24A	0.9600
C2—C17	1.3504 (16)	C24—H24B	0.9600
C2—C3	1.4885 (16)	C24—H24C	0.9600
C3—C4	1.5222 (15)	C25—H25A	0.9600
C4—C6	1.5271 (16)	C25—H25B	0.9600
C4—C5	1.5522 (16)	C25—H25C	0.9600
C4—C8	1.5606 (15)	C26—C31	1.3909 (16)
C5—H5A	0.9700	C26—C27	1.4020 (15)
C5—H5B	0.9700	C27—C28	1.3822 (16)
C6—C26	1.5141 (15)	C27—H27A	0.9300
C6—C7	1.5331 (16)	C28—C29	1.3995 (17)
C6—H6A	0.9800	C29—C30	1.3854 (17)
C7—H7A	0.9700	C30—C31	1.3992 (16)
C7—H7B	0.9700	C30—H30A	0.9300
C8—C16	1.5349 (16)	C31—H31A	0.9300
C8—C9	1.5734 (15)	C32—H32A	0.9600
C9—C10	1.5109 (16)	C32—H32B	0.9600
C10—C11	1.3908 (17)	C32—H32C	0.9600
C10—C15	1.3924 (17)		
			
C9—O2—H1O2	104.8 (15)	C14—C13—H13A	119.8
C22—O4—C24	117.27 (10)	C12—C13—H13A	119.8
C21—O5—H1O5	109.0 (14)	C13—C14—C15	118.26 (13)
C28—O6—C32	116.76 (10)	C13—C14—H14A	120.9
C29—O7—H1O7	106.6 (16)	C15—C14—H14A	120.9
C5—N1—C9	103.00 (9)	C10—C15—C14	121.42 (11)
C5—N1—C1	108.19 (9)	C10—C15—C16	110.47 (10)
C9—N1—C1	114.44 (9)	C14—C15—C16	128.10 (11)
C25—N2—C8	118.12 (10)	O3—C16—C15	127.50 (11)
C25—N2—C7	114.33 (10)	O3—C16—C8	124.03 (11)
C8—N2—C7	109.82 (9)	C15—C16—C8	108.47 (9)
N1—C1—C2	114.38 (9)	C2—C17—C18	129.21 (11)
N1—C1—H1A	108.7	C2—C17—H17A	115.4
C2—C1—H1A	108.7	C18—C17—H17A	115.4
N1—C1—H1B	108.7	C19—C18—C23	118.72 (11)
C2—C1—H1B	108.7	C19—C18—C17	124.51 (11)
H1A—C1—H1B	107.6	C23—C18—C17	116.77 (11)
C17—C2—C3	115.55 (10)	C20—C19—C18	120.02 (12)
C17—C2—C1	125.79 (11)	C20—C19—H19A	120.0
C3—C2—C1	118.11 (10)	C18—C19—H19A	120.0
O1—C3—C2	123.06 (10)	C21—C20—C19	120.93 (12)
O1—C3—C4	122.82 (10)	C21—C20—H20A	119.5
C2—C3—C4	114.01 (9)	C19—C20—H20A	119.5
C3—C4—C6	115.70 (9)	O5—C21—C20	124.24 (11)
C3—C4—C5	107.22 (9)	O5—C21—C22	116.47 (11)
C6—C4—C5	117.65 (9)	C20—C21—C22	119.29 (11)
C3—C4—C8	110.37 (9)	O4—C22—C23	125.26 (11)
C6—C4—C8	104.12 (9)	O4—C22—C21	114.93 (11)
C5—C4—C8	100.54 (9)	C23—C22—C21	119.82 (11)
N1—C5—C4	103.58 (9)	C22—C23—C18	121.21 (11)
N1—C5—H5A	111.0	C22—C23—H23A	119.4
C4—C5—H5A	111.0	C18—C23—H23A	119.4
N1—C5—H5B	111.0	O4—C24—H24A	109.5
C4—C5—H5B	111.0	O4—C24—H24B	109.5
H5A—C5—H5B	109.0	H24A—C24—H24B	109.5
C26—C6—C4	115.37 (9)	O4—C24—H24C	109.5
C26—C6—C7	116.08 (9)	H24A—C24—H24C	109.5
C4—C6—C7	102.67 (9)	H24B—C24—H24C	109.5
C26—C6—H6A	107.4	N2—C25—H25A	109.5
C4—C6—H6A	107.4	N2—C25—H25B	109.5
C7—C6—H6A	107.4	H25A—C25—H25B	109.5
N2—C7—C6	106.40 (9)	N2—C25—H25C	109.5
N2—C7—H7A	110.4	H25A—C25—H25C	109.5
C6—C7—H7A	110.4	H25B—C25—H25C	109.5
N2—C7—H7B	110.4	C31—C26—C27	118.73 (10)
C6—C7—H7B	110.4	C31—C26—C6	123.71 (10)
H7A—C7—H7B	108.6	C27—C26—C6	117.54 (10)
N2—C8—C16	114.64 (9)	C28—C27—C26	120.67 (11)
N2—C8—C4	105.38 (9)	C28—C27—H27A	119.7
C16—C8—C4	115.57 (9)	C26—C27—H27A	119.7
N2—C8—C9	112.43 (9)	O6—C28—C27	124.88 (11)
C16—C8—C9	104.28 (9)	O6—C28—C29	114.88 (10)
C4—C8—C9	104.23 (9)	C27—C28—C29	120.23 (10)
O2—C9—N1	109.40 (9)	O7—C29—C30	119.90 (11)
O2—C9—C10	111.68 (9)	O7—C29—C28	120.46 (10)
N1—C9—C10	114.75 (9)	C30—C29—C28	119.64 (11)
O2—C9—C8	109.24 (9)	C29—C30—C31	119.95 (11)
N1—C9—C8	106.22 (9)	C29—C30—H30A	120.0
C10—C9—C8	105.21 (9)	C31—C30—H30A	120.0
C11—C10—C15	120.26 (11)	C26—C31—C30	120.76 (11)
C11—C10—C9	128.52 (11)	C26—C31—H31A	119.6
C15—C10—C9	111.22 (10)	C30—C31—H31A	119.6
C10—C11—C12	118.26 (13)	O6—C32—H32A	109.5
C10—C11—H11A	120.9	O6—C32—H32B	109.5
C12—C11—H11A	120.9	H32A—C32—H32B	109.5
C11—C12—C13	121.34 (13)	O6—C32—H32C	109.5
C11—C12—H12A	119.3	H32A—C32—H32C	109.5
C13—C12—H12A	119.3	H32B—C32—H32C	109.5
C14—C13—C12	120.43 (13)		
			
C5—N1—C1—C2	51.33 (13)	O2—C9—C10—C15	−122.63 (10)
C9—N1—C1—C2	−62.84 (13)	N1—C9—C10—C15	112.13 (11)
N1—C1—C2—C17	144.18 (12)	C8—C9—C10—C15	−4.23 (12)
N1—C1—C2—C3	−26.93 (15)	C15—C10—C11—C12	−0.28 (19)
C17—C2—C3—O1	30.82 (17)	C9—C10—C11—C12	178.73 (12)
C1—C2—C3—O1	−157.16 (11)	C10—C11—C12—C13	−0.9 (2)
C17—C2—C3—C4	−145.65 (10)	C11—C12—C13—C14	1.3 (2)
C1—C2—C3—C4	26.36 (14)	C12—C13—C14—C15	−0.4 (2)
O1—C3—C4—C6	1.84 (16)	C11—C10—C15—C14	1.15 (18)
C2—C3—C4—C6	178.33 (9)	C9—C10—C15—C14	−178.02 (11)
O1—C3—C4—C5	135.34 (12)	C11—C10—C15—C16	179.93 (11)
C2—C3—C4—C5	−48.17 (12)	C9—C10—C15—C16	0.77 (13)
O1—C3—C4—C8	−116.04 (12)	C13—C14—C15—C10	−0.79 (18)
C2—C3—C4—C8	60.45 (12)	C13—C14—C15—C16	−179.35 (12)
C9—N1—C5—C4	47.67 (10)	C10—C15—C16—O3	−177.64 (12)
C1—N1—C5—C4	−73.85 (11)	C14—C15—C16—O3	1.0 (2)
C3—C4—C5—N1	72.33 (11)	C10—C15—C16—C8	3.18 (13)
C6—C4—C5—N1	−155.22 (9)	C14—C15—C16—C8	−178.14 (11)
C8—C4—C5—N1	−43.03 (10)	N2—C8—C16—O3	−61.39 (15)
C3—C4—C6—C26	77.77 (12)	C4—C8—C16—O3	61.50 (15)
C5—C4—C6—C26	−50.77 (13)	C9—C8—C16—O3	175.26 (11)
C8—C4—C6—C26	−160.93 (9)	N2—C8—C16—C15	117.83 (10)
C3—C4—C6—C7	−154.99 (10)	C4—C8—C16—C15	−119.28 (10)
C5—C4—C6—C7	76.47 (12)	C9—C8—C16—C15	−5.52 (11)
C8—C4—C6—C7	−33.69 (11)	C3—C2—C17—C18	171.74 (11)
C25—N2—C7—C6	121.00 (11)	C1—C2—C17—C18	0.4 (2)
C8—N2—C7—C6	−14.46 (13)	C2—C17—C18—C19	33.3 (2)
C26—C6—C7—N2	156.85 (10)	C2—C17—C18—C23	−146.11 (13)
C4—C6—C7—N2	30.06 (12)	C23—C18—C19—C20	1.62 (18)
C25—N2—C8—C16	−12.37 (14)	C17—C18—C19—C20	−177.73 (11)
C7—N2—C8—C16	121.19 (10)	C18—C19—C20—C21	−0.68 (19)
C25—N2—C8—C4	−140.60 (10)	C19—C20—C21—O5	−179.90 (12)
C7—N2—C8—C4	−7.03 (12)	C19—C20—C21—C22	−0.30 (19)
C25—N2—C8—C9	106.50 (12)	C24—O4—C22—C23	−11.19 (17)
C7—N2—C8—C9	−119.94 (10)	C24—O4—C22—C21	168.71 (11)
C3—C4—C8—N2	150.54 (9)	O5—C21—C22—O4	0.03 (16)
C6—C4—C8—N2	25.76 (11)	C20—C21—C22—O4	−179.60 (11)
C5—C4—C8—N2	−96.49 (9)	O5—C21—C22—C23	179.94 (11)
C3—C4—C8—C16	22.88 (13)	C20—C21—C22—C23	0.30 (17)
C6—C4—C8—C16	−101.90 (10)	O4—C22—C23—C18	−179.43 (11)
C5—C4—C8—C16	135.85 (10)	C21—C22—C23—C18	0.67 (17)
C3—C4—C8—C9	−90.91 (10)	C19—C18—C23—C22	−1.63 (17)
C6—C4—C8—C9	144.31 (8)	C17—C18—C23—C22	177.77 (10)
C5—C4—C8—C9	22.07 (10)	C4—C6—C26—C31	88.03 (13)
C5—N1—C9—O2	85.36 (10)	C7—C6—C26—C31	−32.12 (16)
C1—N1—C9—O2	−157.46 (9)	C4—C6—C26—C27	−90.59 (12)
C5—N1—C9—C10	−148.22 (9)	C7—C6—C26—C27	149.25 (11)
C1—N1—C9—C10	−31.04 (13)	C31—C26—C27—C28	0.63 (17)
C5—N1—C9—C8	−32.44 (10)	C6—C26—C27—C28	179.32 (11)
C1—N1—C9—C8	84.75 (11)	C32—O6—C28—C27	7.83 (19)
N2—C8—C9—O2	1.02 (13)	C32—O6—C28—C29	−172.71 (12)
C16—C8—C9—O2	125.80 (10)	C26—C27—C28—O6	−179.88 (11)
C4—C8—C9—O2	−112.59 (10)	C26—C27—C28—C29	0.70 (18)
N2—C8—C9—N1	118.92 (10)	O6—C28—C29—O7	−0.94 (16)
C16—C8—C9—N1	−116.30 (9)	C27—C28—C29—O7	178.54 (11)
C4—C8—C9—N1	5.30 (11)	O6—C28—C29—C30	179.21 (11)
N2—C8—C9—C10	−119.01 (10)	C27—C28—C29—C30	−1.31 (18)
C16—C8—C9—C10	5.77 (11)	O7—C29—C30—C31	−179.27 (11)
C4—C8—C9—C10	127.37 (9)	C28—C29—C30—C31	0.59 (18)
O2—C9—C10—C11	58.29 (16)	C27—C26—C31—C30	−1.36 (18)
N1—C9—C10—C11	−66.94 (16)	C6—C26—C31—C30	−179.97 (11)
C8—C9—C10—C11	176.69 (12)	C29—C30—C31—C26	0.76 (18)

### Hydrogen-bond geometry (Å, °)

**Table:**

*D*—H···*A*	*D*—H	H···*A*	*D*···*A*	*D*—H···*A*
O5—H1O5···O2^i^	0.89 (2)	1.77 (2)	2.6554 (14)	173 (2)
O7—H1O7···O4^ii^	0.88 (3)	2.13 (3)	2.8720 (13)	143 (2)
O2—H1O2···N2	0.92 (3)	1.91 (2)	2.5987 (13)	131 (2)
C1—H1A···O7^iii^	0.97	2.49	3.3771 (16)	151
C13—H13A···O7^iv^	0.93	2.57	3.3237 (17)	138
C27—H27A···O1	0.93	2.52	3.0998 (15)	121
C32—H32A···O3^v^	0.96	2.40	3.1819 (17)	139

Symmetry codes: (i) −*x*, *y*−1/2, −*z*+1/2; (ii) *x*+1, *y*, *z*; (iii) *x*−1/2, *y*, −*z*+1/2; (iv) *x*−1, *y*, *z*; (v) *x*+1/2, −*y*−1/2, −*z*+1.
